# Inhibiting MiR-33a-3p Expression Fails to Enhance ApoAI-Mediated Cholesterol Efflux in Pro-Inflammatory Endothelial Cells

**DOI:** 10.3390/medicina61020329

**Published:** 2025-02-13

**Authors:** Kun Huang, Achala Pokhrel, Jing Echesabal-Chen, Justin Scott, Terri Bruce, Hanjoong Jo, Alexis Stamatikos

**Affiliations:** 1 Department of Food, Nutrition, and Packaging Sciences, Clemson University, Clemson, SC 29634, USA; kunh@g.clemson.edu (K.H.); achalap@clemson.edu (A.P.); jchen11@clemson.edu (J.E.-C.); 2 Clemson Light Imaging Facility, Clemson University, Clemson, SC 29634, USA; jescott@g.clemson.edu (J.S.); terri@clemson.edu (T.B.); 3 Coulter Department of Biomedical Engineering, Georgia Institute of Technology and Emory University, Atlanta, GA 30322, USA; hjo@emory.edu

**Keywords:** ABC transporters, antagomiR, cardiovascular disease, lipopolysaccharide, reverse cholesterol transport, systemic inflammation

## Abstract

*Background and Objectives*: Atherosclerosis is an inflammatory condition that results in cholesterol accumulating within vessel wall cells. Atherosclerotic cardiovascular disease is the leading cause of mortality worldwide due to this disease being a major contributor to myocardial infarctions and cerebrovascular accidents. Research suggests that cholesterol accumulation occurring precisely within arterial endothelial cells triggers atherogenesis and exacerbates atherosclerosis. Furthermore, inflamed endothelium acts as a catalyst for atherosclerotic development. Therefore, enhancing cholesterol removal specifically in pro-inflammatory endothelial cells may be a potential treatment option for atherosclerosis. While we have previously shown that inhibiting the microRNA guide strand miR-33a-5p within pro-inflammatory endothelial cells increases both ABCA1 expression and apoAI-mediated cholesterol efflux, it is unknown whether inhibiting the miR-33a-3p passenger strand in pro-inflammatory endothelial cells causes similar atheroprotective effects. In this study, this is what we aimed to test. *Materials and Methods*: We used plasmid transfection to knockdown miR-33a-3p expression within cultured pro-inflammatory immortalized mouse aortic endothelial cells (iMAECs). We compared ABCA1 expression and apoAI-mediated cholesterol efflux within these cells to cultured pro-inflammatory iMAECs transfected with a control plasmid. *Results*: The knockdown of miR-33a-3p expression within pro-inflammatory iMAECs resulted in a significant increase in ABCA1 mRNA expression. However, the inhibition of miR-33a-3p did not significantly increase ABCA1 protein expression within pro-inflammatory iMAECs. Moreover, we failed to detect a significant increase in apoAI-mediated cholesterol efflux within pro-inflammatory iMAECs from miR-33a-3p knockdown. *Conclusions*: Our results indicative that the knockdown of miR-33a-3p alone does not enhance ABCA1-dependent cholesterol efflux within pro-inflammatory endothelial cells. To gain any atheroprotective benefit from inhibiting miR-33a-3p within pro-inflammatory endothelium, additional anti-atherogenic strategies would likely be needed in unison.

## 1. Introduction

Despite advances in therapies and treatments, atherosclerotic cardiovascular disease is still the major cause of death globally [1,2]. While the mechanisms of atherosclerosis are complex and not yet fully understood, compelling evidence has demonstrated that inflammation plays a fundamental role in the pathogenesis of atherosclerotic cardiovascular disease [3,4,5]. The pro-inflammatory response that occurs during atherosclerosis is intricately related to endothelial dysfunction, which may initiate atherosclerosis via promoting endothelial barrier permeability that allows cholesterol to invade the sub-endothelial space and enter the intima [6,7,8,9,10]. Endothelial activation also plays a crucial role in both atherogenesis and atherosclerotic progression, since this process facilitates circulatory monocytes to migrate into the intima so that these cells can differentiate into macrophages and engulf intimal cholesterol [11,12,13,14].

Since atherosclerosis ultimately leads to cholesterol accumulation within arteries, the traditional view of atherosclerosis has been that intimal lipid-laden macrophages are primarily responsible for causing atherosclerotic cardiovascular disease [15,16]. However, other cell types within arteries also accumulate cholesterol and appear to play a large role in the pathophysiology of atherosclerosis [17]. For instance, endothelial cells are susceptible to accumulating cholesterol, which is thought to trigger atherosclerotic plaque formation via inducing both endothelial dysfunction and endothelial activation [18,19]. The pro-inflammatory response, which occurs within endothelial cells that accumulate excessive cholesterol, is particularly important to atherogenesis and atherosclerosis progression, since atherosclerotic lesions in humans are shown to largely be confined in locations that exhibit vascular inflammation [20,21,22,23]. Indeed, within the arterial tree, both atheroprone areas and regions with established atherosclerosis are known to demonstrate high levels of endothelial inflammation [24,25,26,27,28]. Hence, removing excess cholesterol precisely from pro-inflammatory endothelial cells may be a potential therapeutic strategy for effectively treating atherosclerosis.

Numerous microRNA have been shown to alter cholesterol, triglyceride, and lipoprotein levels [29,30,31,32,33]. In particular, the microRNA miR-33a is widely recognized as a critical regulator in cholesterol metabolism and homeostasis [34,35]. Indeed, the expression of several genes involved in cholesterol metabolism are regulated by miR-33a [36]. Interestingly, some miR-33a target genes may not be conserved in different species. For example, miR-33a is thought to silence ABCG1 expression in rodents but not in humans [37]. Like other microRNAs, miR-33a is processed into two mature strands, which are miR-33a-5p and miR-33a-3p [38]. Importantly, miR-33a-5p has been more extensively studied and characterized than miR-33a-3p [39]. Interestingly, while miR-33a-5p and miR-33a-3p share certain target genes, data have shown there are also targets unique to these respective mature miR-33a transcripts [40,41,42]. MiR-33a-5p expression has been largely investigated for its role in atheromodulation due to miR-33a-5p effectively silencing the expression of ATP-binding cassette subfamily A, member 1 (ABCA1) [43,44]. Indeed, studies have shown inhibiting miR-33a-5p within endothelial cells and other cell types increases ABCA1 protein expression, resulting in facilitating the removal of intracellular cholesterol via enhancing apoAI-mediated cholesterol efflux [45,46]. While these data suggest inhibiting miR-33a-5p may be atheroprotective, there is virtually no data directly testing whether the inhibition of miR-33a-3p, the other mature strand of miR-33a, may also be atheroprotective. Intriguingly, while miR-33a-3p is not predicted to be a target gene for ABCA1 [46], data does suggest that this microRNA still influences ABCA1 expression within hepatocytes and macrophages through alternative mechanisms via repressing ABCA1 transcriptional activators [47]. However, to our knowledge, there has been no direct testing to precisely determine whether miR-33a-3p expression may be pro-atherogenic within inflamed endothelium through reducing ABCA1-dependent cholesterol efflux in pro-inflammatory endothelial cells.

The purpose of our study is to test the hypothesis that inhibiting miR-33a-3p within pro-inflammatory endothelial cells enhances apoAI-mediated cholesterol efflux via increasing ABCA1 protein expression. In our study, we observed increased miR-33a-3p expression in cultured endothelial cells challenged with LPS to induce a pro-inflammatory state, which corresponds with what occurs to the expression of the other miR-33a mature transcript, miR-33a-5p, within cultured macrophages [48]. When we transfected cultured pro-inflammatory endothelial cells with a plasmid that expresses anti-miR-33a-3p, we detected robust transfection efficiency in these cells in addition to a significant decrease in miR-33a-3p expression. However, while the reduced expression of miR-33a-3p in cultured pro-inflammatory endothelial cells resulted in an increase in ABCA1 expression at the mRNA level, we did not detect an ABCA1 expression increase at the post-transcriptional level and failed to observe enhanced apoAI-mediated cholesterol efflux. Our results indicate that inhibiting miR-33a-3p within inflamed endothelium increases ABCA1 mRNA expression, but to induce an atheroprotective effect, other additional strategies to further increase ABCA1 expression to stimulate apoAI-mediated cholesterol efflux within pro-inflammatory endothelial cells is likely needed.

## 2. Materials and Methods

### 2.1. Cell Culture

Immortalized mouse aortic endothelial cells (iMAECs) were obtained from Dr. Hanjoong Jo’s laboratory [49,50] and primary mouse aortic endothelial cells (pMAECs) were purchased from Cell Biologics Inc. (Chicago, IL, USA). Both iMAECs and pMAECs were cultured in standard growth medium that included high-glucose Dulbecco’s Modified Eagle’s Medium (DMEM; Corning, New York, NY, USA) supplemented with 10% fetal bovine serum (VWR Life Science Seradigm, Radnor, PA, USA) and 1% penicillin/streptomycin (Corning). In addition, antibiotic G418/geneticin (500 ug/mL; VWR Life Science Seradugm) was also added to the medium of iMAECs. Using respective standard growth medium, cells were cultured in 10 cm tissue culture dishes maintained in an incubator set at 37 °C and 5% CO_2_, and medium was replenished approximately 2–3 weekly. To confirm miR-33a-3p expression within iMAECs, we harvested the RNA from these cells and pMAECs cultured in basal conditions as described above and used the total RNA for detection of miR-33a-3p in both cell types.

### 2.2. LPS Challenge in iMAECs

To stimulate pro-inflammatory conditions within iMAECs, we cultured iMAECs in 6-well tissue culture plates, washed cells with PBS, and challenged iMAECs with LPS (10 ng/mL; Sigma-Aldrich, St. Louis, MO, USA) or treated cells with vehicle only [51]. After challenging iMAECs with LPS or treating cells with vehicle only for 24 h, we washed cells with PBS and then either isolated total cellular RNA 24 h later, collected lysates 48 h later, or immediately proceeded with transfecting iMAECs with plasmids.

### 2.3. Plasmid DNA Transfection Within LPS-Challenged iMAECs

Twenty-four hours after inducing inflammation within iMAECs via LPS challenge, we washed iMAECs with PBS and then transfected iMAECs with two different plasmids (System Biosciences, Palo Alto, CA, USA). For control iMAECs, we transfected cells with a plasmid that expresses a non-targeting scrambled shRNA transcript (pScr) that has been validated by us and others to be non-functional [39,45,46,52,53]. For the treatment groups of iMAECs, we transfected these cells with a plasmid that expresses an anti-miR-33a-3p antagomiR for miR-33a-3p (pA3p). We transfected both groups of iMAECs with plasmid DNA via using jetOPTIMUS transfection reagents (Polyplus, New York, NY, USA) as previously described [54], and both plasmids described above also contain an expression cassette that encodes GFP protein. After plasmid DNA transfections, we either harvested total cellular RNA 24 h later, prepared lysates 48 h later, or performed flow cytometry 48 h later.

### 2.4. End-Point RT-PCR for Small RNA

We first isolated total RNA from either iMAECs or pMAECs by using a Zymo Research Direct-zol total RNA column-purification kit (Irvine, CA, USA) [55]. We converted the purified total cellular RNA into cDNA by using a Takara Mir-X miRNA first-strand cDNA synthesis kit (San Jose, CA, USA) [39]. We then used the newly synthesized cDNA as template for end-point PCR to amplify miR-33a-3p by utilizing a TB Green Advantage qPCR Pre-mix Kit (Takara). After amplification of miR-33a-3p, the PCR products remained either unexposed to restriction enzymes, treated with the restriction enzyme *Bsr*DI, or digested with the restriction enzyme *Tsp*RI (New England Biolabs, Ipswich, MA, USA). We subsequently analyzed all of the amplicons and digested fragments by using TBE agarose gel electrophoresis technology coupled with a GelDoc imager (Analytik Jena US, Upland, CA, USA) [56]. To detect U6, scrambled shRNA, and anti-miR-33a-3p transcripts within the total cellular RNA collected from transfected iMAECs, we utilized the same procedures for end-point RT-PCR as described above, with all PCR products remaining untreated. The primers used for these PCR reactions are listed within Table 1.

### 2.5. RT-qPCR (Small RNA and mRNA)

To quantify miR-33a-3p and anti-miR-33a-3p expression within total RNA extracted from iMAECs, we initially performed the same protocols outlined above for end-point RT-PCR as previously described [39]. To analyze small RNA gene expression, we quantified our qPCR reactions by using an Analytik Jena US qTOWER^3^ G touch qPCR instrument [57]. To measure mRNA expression of ABCA1 and Niemann–Pick type C1 (NPC1), we first converted total RNA extracted from cultured iMAECs into cDNA via using a qScript cDNA Ultra SuperMix Kit (Quantabio, Beverly, MA, USA) [58]. We conducted our qPCR reactions using this newly synthesized cDNA as template along with a PerfeCTa SYBR Green FastMix Kit (Quantabio) [51] and quantified these qPCR reactions as described above. To normalize gene expression, we used U6 as the reference “housekeeping” for small RNA expression and utilized GAPDH as the reference/housekeeping gene for mRNA expression. All primers used during our qPCR reactions are shown in Table 1 and gene expression levels were calculated by performing the ΔΔ^CT^ method [59].

### 2.6. Plasmid Transfection Efficiency Within iMAECs

We initially challenged iMAECs with LPS as described above and then either mock-transfected cells or transfected iMAECs with either pScr or pA3p as previously described. After 48 h of transfection, we washed iMAECs with PBS, trypsinized cells, and resuspended iMAECs with 1 mL of PBS before pelleting cells via centrifugation. We then decanted supernatant and resuspended the pelleted cells with 1 mL of fresh PBS to use for FACS analysis. Briefly, iMAECs were analyzed by using a Bio-Rad S3e Cell Sorter/Cytometer (Hercules, CA, USA) and Bio-Rad Prosort acquisition software (v1.6.0.12). Mock-transfected iMAECs were compared with plasmid DNA transfected iMAECs to verify and adjust for background fluorescence signal. Ten-thousand events were gated onto an FSC/SSC plot to exclude cellular debris and retain a viable cell population. This population was then gated to allow singlet events to pass through the detector. From those singlets, GFP^+^ cells were identified by using a 525/30 nm filter and recorded to determine the percentage of GFP^+^ iMAECs to the total number of intact, viable cells.

### 2.7. Western Blotting

We conducted immunoblotting as previously described [54,60]. Briefly, we collected cultured iMAEC lysates using RIPA lysis buffer containing mammalian protease inhibitors (VWR Life Science) and quantified protein concentrations within these lysates by using a BCA assay kit (BioVison, Milpitas, CA, USA). Using equal protein mass, we separated proteins within lysates via SDS-PAGE and transferred the separated proteins onto PVDF membranes (Merck Millipore Ltd., Burlington, MA, USA). After blocking PVDF membranes in blocking buffer, we incubated the PVDF membranes with the following primary antibodies: anti-ABCA1 (1:1000 dilution, sc-58219; Santa Cruz Biotechnology, Dallas, TX, USA); anti-NPC1 (1:2500 dilution, NB400-148, Novus Biologicals, Littleton, CO, USA); anti-VCAM-1 (1:2000 dilution, sc-13160; Santa Cruz Biotechnology); anti-GAPDH (1:2500 dilution; sc-365062; Santa Cruz Biotechnology). After primary antibody incubation, we incubated the PVDF membranes with either HRP-conjugated goat anti-mouse IgG secondary antibody (1:10,000 dilution, AP181P; Sigma-Aldrich, St. Louis, MO, USA) or HRP-conjugated goat anti-rabbit IgG secondary antibody (1:10,000 dilution, HAF008; Novus Biologicals, Littleton, CO, USA). We incubated PVDF membranes with ECL substrate (Immobilon ECL Ultra Western HRP Substrate; MiliporeSigma, Billerica, MA, USA) after secondary antibody incubation, visualized proteins using a ChemiDoc imager (Analytik Jena US), and quantified protein expression via utilizing version 1.53a NIH ImageJ software [61].

### 2.8. Cholesterol Efflux

We measured apoAI-mediated cholesterol efflux by adapting our previously published methods [52,62]. In these cholesterol efflux assays, we first challenged iMAECs with LPS (10 ng/mL) for 24 h to induce a pro-inflammatory state. We then transfected LPS-challenged iMAECs with either pScr or pA3p for 24 h as described above. Post-plasmid DNA transfection, we washed iMAECs with PBS and loaded cells with [3H] cholesterol (1ulCi/mL; PerkinElmer, Waltham, MA, USA) diluted in high-glucose, serum-free DMEM supplemented with 1% penicillin/streptomycin and 2 mg/mL of fatty acid-free bovine serum albumin (Sigma-Aldrich). After 24 h, we washed iMAECs with PBS and incubated cells with either apoAI (5 ug/mL; Academy Bio-Medical Company, Houston, TX, USA) or vehicle only for 24 h. The serum-free medium used during apoAI/vehicle exposure was high-glucose DMEM supplemented with 1% penicillin/streptomycin and 2 mg/mL of fatty acid-free bovine serum albumin. After apoAI/vehicle treatments, we collected both medium and iMAECs to count [3H] in cells/medium by using a PerkinElmer Tri-Carb 4910TR liquid scintillation counter (Waltham, MA, USA). From these measurements, we calculated apoAI-mediated cholesterol efflux as previously described [45,63].

### 2.9. Statistical Analyses

All statistical testing conducting involved 2 groups. Normality was tested by using a Shapiro–Wilk test. In the event that normality was not assumed, we performed a Mann–Whitney rank-sum test. Equal variance was tested by using a Brown–Forsythe test. In the scenario that equal variance assumptions were violated, we performed Welch’s *t*-test. When both normality and equal variance were assumed, we performed Student’s *t*-test. We conducted all statistical analyses by using SigmaPlot (Systat Software Inc. v14.0, Chicago, IL, USA) and we set the level of statistical significance at *p* < 0.05.

## 3. Results

### 3.1. Pro-Inflammatory Endothelial Cells Demonstrate Increased MiR-33a-3p Expression

**Figure 1:**
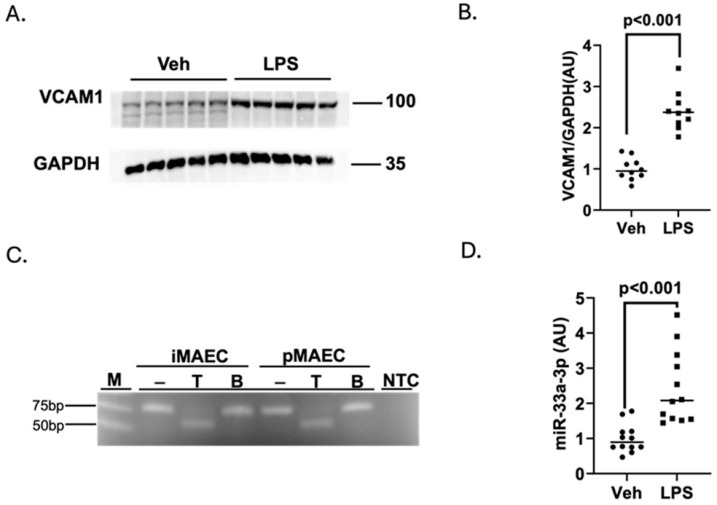
Challenging iMAECs with LPS induces VCAM-1 protein expression and leads to increased miR-33a-3p expression. (**A**,**B**). Representatives immunoblot (**A**) and densitometry (**B**) of vehicle (Veh)-treated and LPS-challenged iMAECs for measuring VCAM-1 protein with GAPDH used as the housekeeping protein. Data points (**B**) are from two independent experiments with five biological replicates used in each experiment. (**C**) End-point RT-PCR and restriction digest of PCR products assessed by TBE agarose gel electrophoresis to detect miR-33a-3p. MiR-33a-3p cDNA contains one *Tsp*RI restriction site but lacks *Bsr*DI restriction sites. M, DNA marker; NTC, non-template control PCR reaction; minus (−) indicates non-digested amplicons; T, *Tsp*RI-digested amplicons; B, *Bsr*DI-digested amplicons; pMAEC total RNA was used as template for positive technical control end-point RT-PCR and restriction digestion reactions. (**D**) MiR-33a-3p expression measured in vehicle-treated and LPS-challenged iMAECs via RT-qPCR. Data points show two independent experiments with six biological replicates used for both experiments. (**A**) Size markers are in kDa; (**B**,**D**) bars are group means. AU = arbitrary units.

Since we want to utilize pro-inflammatory endothelial cells in our downstream experiments, we first challenged iMAECs with the endotoxin LPS to attempt to induce a pro-inflammatory state within these cells. Incubating iMAECs with LPS resulted in a significant increase in protein expression for the endothelial cell adhesion molecule VCAM-1 (Figure 1A,B), which indicates a pro-inflammatory state within these cells [64,65]. In our previous studies, we characterized iMAECs to confirm that they express miR-33a-5p and ABCA1 protein and effectively efflux cholesterol to apoAI [50,52]. However, since we wanted to manipulate the expression of miR-33a-3p in our current work, we also wanted to confirm that iMAECs express this miR-33a mature strand as well. Based on our results, which combined end-point RT-PCR with restriction digestion, we were able to confidently detect miR-33a-3p expression within iMAECs (Figure 1C). We next confirmed whether challenging iMAECs with LPS alters miR-33a-3p expression. Interestingly, we did detect an increase in miR-33a-3p expression within iMAECs challenged with LPS (Figure 1D), which is a similar finding to the increase observed for miR-33a-5p expression within pro-inflammatory cultured macrophages [48].

### 3.2. LPS-Challenged iMAECs Exhibit Robust Plasmid DNA Transfection Efficiency

**Figure 2:**
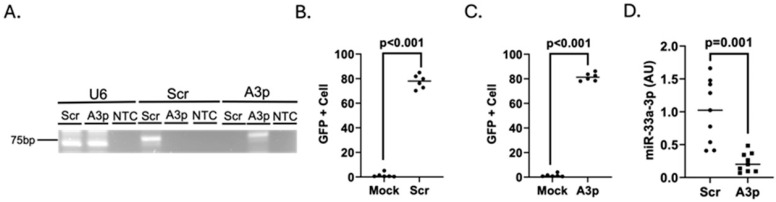
Transfecting pro-inflammatory endothelial cells with pA3p decreases miR-33a-3p expression. (**A**) End-point RT-PCR coupled with TBE agarose gel electrophoresis was used for detecting transgenic anti-miRs within LPS-challenged iMAECs transfected with either pScr of pA3p. Top labels: U6, positive control PCR reactions to detect U6; Scr, PCR reactions for detecting scrambled anti-miR; A3p, PCR reactions for detecting anti-miR-33a-3p. Bottom labels: Scr, cDNA from pScr-transfected iMAECs; A3p, cDNA from pA3p-transfected iMAECs; NTC, non-template control. (**B**,**C**) Percent of intact GFP^+^ cells detected via flow cytometry counted from LPS-challenged iMAECs transfected with either pScr (**B**) or pA3p (**C**) versus respective mock-transfected, LPS-challenged iMAECs. Both pScr and pA3p express GFP. Data points indicate two independent treatments with three biological replicates per respective treatment and bars are group means. (**D**) MiR-33a-3p expression measured via RT-qPCR in LPS-challenged iMAECs transfected with either pScr (Scr) or pA3p (A3p). Data points indicate three independent experiments with three biological replicates per respective experiment. Bars are group means and AU indicates arbitrary units.

For us to reliably determine whether miR-33a-3p inhibition is capable of upregulating ABCA1 protein expression in pro-inflammatory endothelium, we first needed to confirm that we were successful with introducing sufficient levels of pA3p into these cells. The rationale for this is because there are challenges with transfecting certain immortalized cell lines, and stimulating inflammation within cultured cells can decrease transfection efficiency [66,67,68,69,70,71,72]. To initially assess transfection efficiency, we used end-point RT-PCR to confirm respective transgenic transcripts were expressed within LPS-challenged iMAECs transfected with either pA3p or pScr (Figure 2A). To calculate transfection efficiency, we performed flow cytometry to determine the percentage of LPS-challenged iMAECs transfected with either pScr or pA3p and observed high transfection efficiency from both plasmids (Figure 2B,C). We next examined the impact of miR-33a-3p expression within LPS-challenged iMAECs transfected with pA3p when compared to LPS-challenged iMAECs transfected with pScr (Figure 2D). In these experiments, we detected a significant reduction in miR-33a-3p expression in cells transfected with pA3p plasmid DNA. These results show that transfecting cultured pro-inflammatory endothelial cells with pA3p successfully inhibits miR-33a-3p expression within these cells.

### 3.3. Transfecting Pro-Inflammatory iMAECs with pA3p Upregulates NPC1 Expression

**Figure 3:**
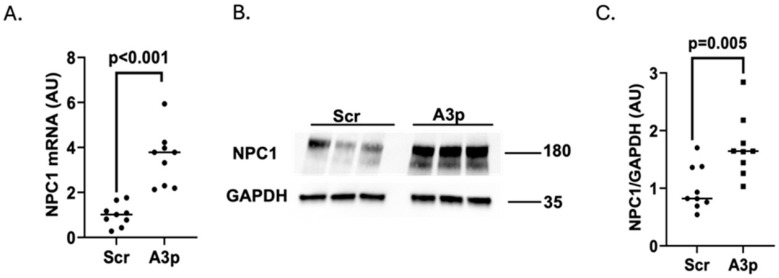
Using plasmid transfection to inhibit miR-33a-3p expression within pro-inflammatory endothelial cells increases NPC1 expression. (**A**–**C**) LPS-challenged iMAECs were transfected with either pScr (Scr) or pA3p (A3p). (**A**) NPC1 mRNA expression assessed by RT-qPCR. (**B**,**C**) Representative Western blot (**B**) and densitometry (**C**) for analysis of NPC1 protein expression using GAPDH as the loading control. (**A**,**C**) Data points indicate three independent treatments with three biological replicates per respective treatment and bars are group means; AU, arbitrary units. (**B**) Size markers are in kDa.

One of the most established and recognized target genes for miR-33a-3p is NPC1 [30,41]. Therefore, before analyzing whether the inhibition of miR-33a-3p in pro-inflammatory endothelial cells enhances ABCA1-dependent cholesterol efflux, we first wanted to confirm that pA3p transfection within LPS-challenged iMAECs increases the expression of NPC1 in these cells. When we compared NPC1 expression in LPS-challenged iMAECs transfected with pA3p versus pScr, we observed an increased expression of NPC1 at both the mRNA (Figure 3A) and post-transcriptional level (Figure 3B,C) within pro-inflammatory iMAECs transfected with pA3p plasmids. From these findings, we conclude that transfecting pro-inflammatory endothelial cells with pA3p increases the expression of the well-established miR-33a-3p target gene NPC1, and this demonstrates potential in increasing the expression of other possible miR-33a-3p target genes.

### 3.4. Transfecting Pro-Inflammatory iMAECs with pA3p Fails to Enhance ABCA1-Dependent Cholesterol Efflux

**Figure 4:**
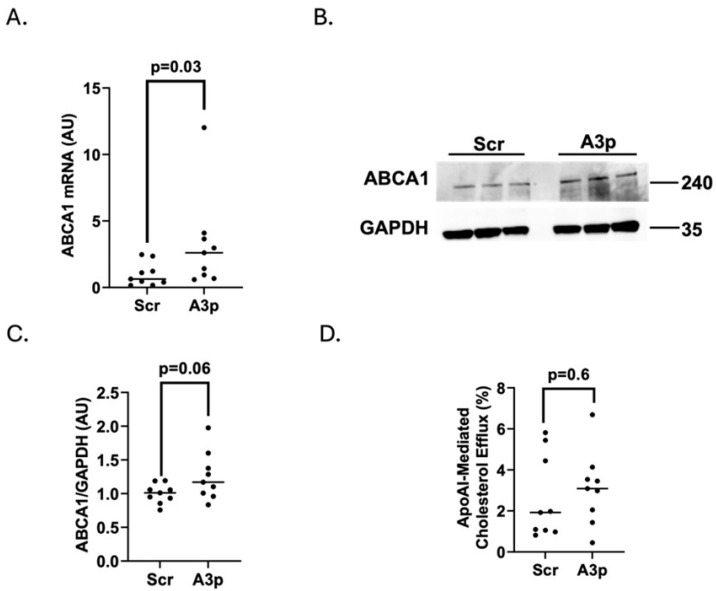
Using plasmid transfection to inhibit miR-33a-3p expression within pro-inflammatory endothelial cells does not increase apoAI-mediated cholesterol efflux. (**A**–**C**) iMAECs were challenged with LPS then transfected with either pScr (Scr) or pA3p (A3p). (**A**) ABCA1 mRNA expression analyzed via RT-qPCR. (**B**,**C**) Representative immunoblot (**B**) and densitometry (**C**) to assess ABCA1 protein levels with GAPDH used as a housekeeping protein. (**D**) Percent cellular cholesterol efflux measured after exposing cells to the apoAI cholesterol acceptor. (**A**,**C**) AU = arbitrary units; (**A**,**C**,**D**) data points indicate three independent experiments with three biological replicates in each experiment and bars showing group means; (**B**) size markers are in kDa.

While we have already shown that inhibiting miR-33a-5p within pro-inflammatory endothelial cells via plasmid transfection increases ABCA1-dependent cholesterol efflux [52], it is unknown what effect miR-33a-3p inhibition has on ABCA1 expression and apoAI-mediated cholesterol efflux within pro-inflammatory endothelial cells. Thus, we first measured ABCA1 mRNA expression within LPS-challenged iMAECs transfected with either pA3p or pScr and detected a significant increase in ABCA1 mRNA levels within pA3p-transfected pro-inflammatory iMAECs (Figure 4A). However, when we analyzed ABCA1 expression at the post-transcriptional level, we only observed a trend towards increased ABCA1 protein expression (*p* = 0.06) when we compared LPS-challenged iMAECs transfected with pA3p to LPS-challenged iMAECs transfected with pScr (Figure 4B,C). However, the changes observed in ABCA1 expression within pA3p-transfected pro-inflammatory iMAECs were shown to have no biological impact on apoAI-mediated cholesterol efflux (Figure 4D). Our results show that while our transfection methods used for delivering pA3p to pro-inflammatory endothelial cells increases mRNA expression within these cells, this delivery method failed to significantly increase apoAI-mediated cellular cholesterol efflux.

## 4. Discussion

In this study, we wanted to assess whether inhibiting miR-33a-3p within pro-inflammatory ECs in vitro enhances apoAI-mediated cholesterol efflux via increasing ABCA1 protein expression. We first identified that iMAECs expresses miR-33a-3p, making this immortalized EC line suitable for our studies, as we already showed iMAECs express functional ABCA1, capable of effluxing cholesterol to apoAI sufficiently [50,52]. Interestingly, when we challenged iMAECs with LPS, we observed increased miR-33a-3p expression, which parallels what occurs with miR-33a-5p expression in cultured macrophages exhibiting a pro-inflammatory state [48]. When we utilized plasmid DNA transfection for inhibiting miR-33a-3p expression in pro-inflammatory iMAECs, we were successful at reducing the expression of this microRNA, which resulted in a significant increase in ABCA1 mRNA expression. However, inhibiting miR-33a-3p expression within pro-inflammatory iMAECs did not significantly increase ABCA1 protein expression. Since ABCA1 protein expression was not robustly upregulated within pro-inflammatory iMAECs, as expected, we did not observe enhanced apoAI-mediated cholesterol efflux within these cells.

Several microRNA have been proposed to be pro-atherogenic [73,74,75,76,77,78,79,80], but miR-33a is considered to be the most characterized [36]. Indeed, it has been ~15 years since miR-33a has been identified to be a potential pro-atherogenic driver [40,46]. Since then, miR-33a has arguably been the most extensively studied microRNA within the context of atherosclerosis [36]. Though we and other laboratories have extensively evaluated the atherogenic effects of miR-33a in several cell types [39,44,45,51,52,55,81,82,83], these studies have predominantly focused on miR-33a-5p, and scant data have been published on assessing whether miR-33a-3p plays an atherogenic role in humans [47]. While earlier studies showed promise that inhibiting miR-33a-(5p) systemically may have atheroprotective benefits [40,46,84], other data suggests that the systemic inhibition of this microRNA may actually exhibit pro-atherogenic properties [85,86]. The perplexity of these conflicting findings has led to the proposal that miR-33a inhibition within the liver in vivo may actually lead to an atherogenic environment [43,44]. However, even this has been recently questioned [81,87]. While the atheromodulatory effects surrounding miR-33a expression appear convoluted, there seems to be no disputing that inhibiting miR-33a expression precisely in macrophages results in atheroprotection [88,89,90]. Much of this published work outlined above involves inhibiting miR-33a-5p but not miR-33a-3p, and, to our knowledge, there are no published data we are aware of that involve directly testing the atheromodulatory impact of inhibiting miR-33a-3p within pro-inflammatory ECs, which led us to pursue this work.

An inflamed endothelium drives atherosclerosis, and cholesterol accumulation within ECs triggers vascular inflammation [91,92,93,94]. Since we and others have shown that EC ABCA1 expression is atheroprotective [63,95,96], we wanted to identify novel methods to upregulate ABCA1 expression precisely in pro-inflammatory ECs. While our laboratory has previously shown that inhibiting miR-33a-5p via plasmid transfection within pro-inflammatory EC enhances ABCA1-dependent cholesterol efflux [52], we wanted to possibly discover if inhibiting miR-33a-3p in these cells may lead to similar atheroprotective effects. Indeed, if successful, the inhibition of both miR-33a-5p and miR-33a-3p may result in added synergistic properties beyond simply inhibiting one of these microRNAs. However, in our experiments, we failed to detect an increase in ABCA1-dependent cholesterol efflux within pro-inflammatory ECs via miR-33a-3p inhibition.

In this study, we want to highlight some potential limitations. One possible limitation is using iMAECs instead of pMAECs in our experiments when assessing the biological impact of miR-33a-3p inhibition. While we rigorously characterized this cell line previously to demonstrate it is an appropriate EC line to use in our proposed experiments [49,50,52] and confirmed miR-33a-3p expression within iMAECs in this study, caution should still be warranted when using immortalized cell lines over primary cells [97]. Another potential limitation is using LPS to induce a pro-inflammatory state within iMAECs. While we have been successful using this strategy in our previously published work involving iMAECs and miR-33a-5p manipulation [52], LPS may still exhibit toxic properties that might mask any atheroprotective benefits from miR-33a-3p inhibition within ECs. Thus, future studies may include assessing any atheromodulatory properties miR-33a-3p expression exhibits within pro-inflammatory ECs that are challenged with other types of inflammatory stimuli like TNF-α [98]. Another possible concern is utilizing plasmid DNA transfection to introduce anti-miR-33a-3p instead of using viral particles. While this may not be a drawback due to the robust transfection efficiency observed in our study that resulted in downregulating mRNA and protein expression of the miR-33a-3p target NPC1 [30,41], viral vectors are still considered superior to plasmid transfections when introducing transgenes [66]. For instance, studies have shown that helper-dependent adenoviruses demonstrate exceptional transduction efficiency within ECs [45,63]; thus, future studies can repeat our work by replacing plasmids with helper-dependent adenoviral vectors to directly test whether introducing anti-miR-33a-3p using these viral particles results in any atheroprotective effects. While our report shows that miR-33a-3p does not appear to enhance ABCA1-dependent cholesterol efflux within pro-inflammatory ECs, there may be alternative atheroprotective effects demonstrated by miR-33a-3p. For instance, miR-33a appears to play a role in autophagy [90], so future studies should examine whether miR-33a-3p expression alters autophagy in pro-inflammatory ECs. Lastly, there may be an issue with the selected time duration used in our experiments when measuring end-points. While we followed a similar experimental design that was successful when we inhibited miR-33a-5p within pro-inflammatory ECs [52], it is certainly possible that performing time-course experiments may uncover that miR-33a-3p inhibition within pro-inflammatory ECs has the potential to enhance apoAI-mediated cholesterol efflux.

In conclusion, our study found that the suppression of miR-33a-3p within pro-inflammatory endothelial cells did not increase apoAI-mediated cholesterol efflux. The failure to enhance apoAI-mediated cholesterol efflux in these cells is likely due to not observing a significant increase in ABCA1 protein expression. However, in our studies, the significant decrease in miR-33a-3p expression within pro-inflammatory ECs did increase the expression of ABCA1 in the mRNA. While this increase in mRNA expression may be enticing, from our study, inhibiting miR-33a-3p expression within pro-inflammatory ECs may likely only be utilized as an adjunct strategy combined with other compounds, such as LXR agonists and cAMP analogs, which are already established to increase ABCA1 activity, ABCA1 protein expression, and ultimately apoAI-mediated cholesterol efflux [50,99,100,101].

## Figures and Tables

**Table 1:** Primer pairs.

Target	Sequence (5′-3′)
NPC1	forward: TGTTTGGTATGGAGAGTGTGGA
	reverse: GTCACAGCAGAGACTGACATTG
ABCA1	forward: AAAACCGCAGACATCCTTCAG
	reverse: CATACCGAAACTCGTTCACCC
GAPDH	forward: AGGTCGGTGTGAACGGATTTG
	reverse: GGGGTCGTTGATGGCAACA
U6	forward: GGAACGATACAGAGAAGATTAGC
	reverse: TGGAACGCTTCACGAATTTGCG
MiR-33a-3p	forward: CAATGTTTCCACAGTGCATCA
Anti-miR-33a-3p	forward: GTGATGCACTGTGGAAACATTG
Scrambled Anti-miR	forward: TAAGGTTAAGTCGCCCTCGC
Universal Small RNA	reverse: Proprietary (Takara)

## Data Availability

All data included in this study are provided within the manuscript.

## References

[1] Hetherington I., Totary-Jain H. (2022). Anti-atherosclerotic therapies: Milestones, challenges, and emerging innovations. Mol. Ther..

[2] Mathers C.D., Loncar D. (2006). Projections of global mortality and burden of disease from 2002 to 2030. PLoS Med..

[3] Cockerill G., Xu Q., Fitridge R., Thompson M. (2011). Atherosclerosis. Mechanisms of Vascular Disease: A Reference Book for Vascular Specialists.

[4] Bjorkegren J.L.M., Lusis A.J. (2022). Atherosclerosis: Recent developments. Cell.

[5] Taleb S. (2016). Inflammation in atherosclerosis. Arch. Cardiovasc. Dis..

[6] Mani A.M., Chattopadhyay R., Singh N.K., Rao G.N. (2018). Cholesterol crystals increase vascular permeability by inactivating SHP2 and disrupting adherens junctions. Free Radic. Biol. Med..

[7] Gimbrone M.A., Garcia-Cardena G. (2016). Endothelial Cell Dysfunction and the Pathobiology of Atherosclerosis. Circ. Res..

[8] Higashi Y. (2023). Endothelial Function in Dyslipidemia: Roles of LDL-Cholesterol, HDL-Cholesterol and Triglycerides. Cells.

[9] Mundi S., Massaro M., Scoditti E., Carluccio M.A., van Hinsbergh V.W.M., Iruela-Arispe M.L., De Caterina R. (2018). Endothelial permeability, LDL deposition, and cardiovascular risk factors-a review. Cardiovasc. Res..

[10] Medina-Leyte D.J., Zepeda-Garcia O., Dominguez-Perez M., Gonzalez-Garrido A., Villarreal-Molina T., Jacobo-Albavera L. (2021). Endothelial Dysfunction, Inflammation and Coronary Artery Disease: Potential Biomarkers and Promising Therapeutical Approaches. Int. J. Mol. Sci..

[11] Medrano-Bosch M., Simon-Codina B., Jimenez W., Edelman E.R., Melgar-Lesmes P. (2023). Monocyte-endothelial cell interactions in vascular and tissue remodeling. Front. Immunol..

[12] Hernandez G.E., Iruela-Arispe M.L. (2020). The many flavors of monocyte/macrophage--endothelial cell interactions. Curr. Opin. Hematol..

[13] Schubert S.Y., Benarroch A., Monter-Solans J., Edelman E.R. (2010). Monocyte activation state regulates monocyte-induced endothelial proliferation through Met signaling. Blood.

[14] Jebari-Benslaiman S., Galicia-Garcia U., Larrea-Sebal A., Olaetxea J.R., Alloza I., Vandenbroeck K., Benito-Vicente A., Martin C. (2022). Pathophysiology of Atherosclerosis. Int. J. Mol. Sci..

[15] Theofilis P., Oikonomou E., Tsioufis K., Tousoulis D. (2023). The Role of Macrophages in Atherosclerosis: Pathophysiologic Mechanisms and Treatment Considerations. Int. J. Mol. Sci..

[16] Moore K.J., Sheedy F.J., Fisher E.A. (2013). Macrophages in atherosclerosis: A dynamic balance. Nat. Rev. Immunol..

[17] Tabas I., Garcia-Cardena G., Owens G.K. (2015). Recent insights into the cellular biology of atherosclerosis. J. Cell Biol..

[18] Tete S., Tripodi D., Rosati M., Conti F., Maccauro G., Saggini A., Salini V., Cianchetti E., Caraffa A., Antinolfi P. (2012). Endothelial cells, cholesterol, cytokines, and aging. Int. J. Immunopathol. Pharmacol..

[19] Jiang H., Zhou Y., Nabavi S.M., Sahebkar A., Little P.J., Xu S., Weng J., Ge J. (2022). Mechanisms of Oxidized LDL-Mediated Endothelial Dysfunction and Its Consequences for the Development of Atherosclerosis. Front. Cardiovasc. Med..

[20] Kwak B.R., Back M., Bochaton-Piallat M.L., Caligiuri G., Daemen M.J., Davies P.F., Hoefer I.E., Holvoet P., Jo H., Krams R. (2014). Biomechanical factors in atherosclerosis: Mechanisms and clinical implications. Eur. Heart J..

[21] Tamargo I.A., Baek K.I., Kim Y., Park C., Jo H. (2023). Flow-induced reprogramming of endothelial cells in atherosclerosis. Nat. Rev. Cardiol..

[22] Nigro P., Abe J., Berk B.C. (2011). Flow shear stress and atherosclerosis: A matter of site specificity. Antioxid. Redox Signal.

[23] Pahwa R., Jialal I. (2023). Disclosure: Ishwarlal Jialal declares no relevant financial relationships with ineligible companies. Atherosclerosis.

[24] Davies P.F., Civelek M., Fang Y., Fleming I. (2013). The atherosusceptible endothelium: Endothelial phenotypes in complex haemodynamic shear stress regions in vivo. Cardiovasc. Res..

[25] Gusev E., Sarapultsev A. (2023). Atherosclerosis and Inflammation: Insights from the Theory of General Pathological Processes. Int. J. Mol. Sci..

[26] Botts S.R., Fish J.E., Howe K.L. (2021). Dysfunctional Vascular Endothelium as a Driver of Atherosclerosis: Emerging Insights into Pathogenesis and Treatment. Front. Pharmacol..

[27] Li H., Zhou W.Y., Xia Y.Y., Zhang J.X. (2022). Endothelial Mechanosensors for Atheroprone and Atheroprotective Shear Stress Signals. J. Inflamm. Res..

[28] Green J.P., Souilhol C., Xanthis I., Martinez-Campesino L., Bowden N.P., Evans P.C., Wilson H.L. (2018). Atheroprone flow activates inflammation via endothelial ATP-dependent P2X7-p38 signalling. Cardiovasc. Res..

[29] Aryal B., Singh A.K., Rotllan N., Price N., Fernandez-Hernando C. (2017). MicroRNAs and lipid metabolism. Curr. Opin. Lipidol..

[30] Citrin K.M., Fernandez-Hernando C., Suarez Y. (2021). MicroRNA regulation of cholesterol metabolism. Ann. N. Y. Acad. Sci..

[31] Fernandez-Tussy P., Ruz-Maldonado I., Fernandez-Hernando C. (2021). MicroRNAs and Circular RNAs in Lipoprotein Metabolism. Curr. Atheroscler. Rep..

[32] Wagschal A., Najafi-Shoushtari S.H., Wang L., Goedeke L., Sinha S., deLemos A.S., Black J.C., Ramirez C.M., Li Y., Tewhey R. (2015). Genome-wide identification of microRNAs regulating cholesterol and triglyceride homeostasis. Nat. Med..

[33] Xiang Y., Mao L., Zuo M.L., Song G.L., Tan L.M., Yang Z.B. (2022). The Role of MicroRNAs in Hyperlipidemia: From Pathogenesis to Therapeutical Application. Mediators Inflamm..

[34] Rottiers V., Naar A.M. (2012). MicroRNAs in metabolism and metabolic disorders. Nat. Rev. Mol. Cell Biol..

[35] Ono K. (2016). Functions of microRNA-33a/b and microRNA therapeutics. J. Cardiol..

[36] Sidorkiewicz M. (2023). Is microRNA-33 an Appropriate Target in the Treatment of Atherosclerosis?. Nutrients.

[37] Fernandez-Hernando C., Baldan A. (2013). MicroRNAs and Cardiovascular Disease. Curr. Genet. Med. Rep..

[38] Tanashyan M.M., Shabalina A.A., Kuznetsova P.I., Raskurazhev A.A. (2023). miR-33a and Its Association with Lipid Profile in Patients with Carotid Atherosclerosis. Int. J. Mol. Sci..

[39] Oladosu O., Chin E., Barksdale C., Powell R.R., Bruce T., Stamatikos A. (2024). Inhibition of miR-33a-5p in Macrophage-like Cells In Vitro Promotes apoAI-Mediated Cholesterol Efflux. Pathophysiology.

[40] Najafi-Shoushtari S.H., Kristo F., Li Y., Shioda T., Cohen D.E., Gerszten R.E., Naar A.M. (2010). MicroRNA-33 and the SREBP host genes cooperate to control cholesterol homeostasis. Science.

[41] Han S.Y., Han H.B., Tian X.Y., Sun H., Xue D., Zhao C., Jiang S.T., He X.R., Zheng W.X., Wang J. (2016). MicroRNA-33a-3p suppresses cell migration and invasion by directly targeting PBX3 in human hepatocellular carcinoma. Oncotarget.

[42] Gao C., Wei J., Tang T., Huang Z. (2020). Role of microRNA-33a in malignant cells. Oncol. Lett..

[43] Naar A.M. (2013). Anti-atherosclerosis or No Anti-atherosclerosis: That is the miR-33 question. Arterioscler. Thromb. Vasc. Biol..

[44] Naar A.M. (2018). miR-33: A Metabolic Conundrum. Trends Endocrinol. Metab..

[45] Stamatikos A., Knight E., Vojtech L., Bi L., Wacker B.K., Tang C., Dichek D.A. (2020). Exosome-Mediated Transfer of Anti-miR-33a-5p from Transduced Endothelial Cells Enhances Macrophage and Vascular Smooth Muscle Cell Cholesterol Efflux. Hum. Gene Ther..

[46] Rayner K.J., Suarez Y., Davalos A., Parathath S., Fitzgerald M.L., Tamehiro N., Fisher E.A., Moore K.J., Fernandez-Hernando C. (2010). MiR-33 contributes to the regulation of cholesterol homeostasis. Science.

[47] Goedeke L., Vales-Lara F.M., Fenstermaker M., Cirera-Salinas D., Chamorro-Jorganes A., Ramirez C.M., Mattison J.A., de Cabo R., Suarez Y., Fernandez-Hernando C. (2013). A regulatory role for microRNA 33* in controlling lipid metabolism gene expression. Mol. Cell Biol..

[48] Mao M., Lei H., Liu Q., Chen Y., Zhao L., Li Q., Luo S., Zuo Z., He Q., Huang W. (2014). Effects of miR-33a-5P on ABCA1/G1-mediated cholesterol efflux under inflammatory stress in THP-1 macrophages. PLoS ONE.

[49] Ni C.W., Kumar S., Ankeny C.J., Jo H. (2014). Development of immortalized mouse aortic endothelial cell lines. Vasc. Cell.

[50] Huang K., Jo H., Echesabal-Chen J., Stamatikos A. (2021). Combined LXR and RXR Agonist Therapy Increases ABCA1 Protein Expression and Enhances ApoAI-Mediated Cholesterol Efflux in Cultured Endothelial Cells. Metabolites.

[51] Echesabal-Chen J., Huang K., Vojtech L., Oladosu O., Esobi I., Sachdeva R., Vyavahare N., Jo H., Stamatikos A. (2023). Constructing Lipoparticles Capable of Endothelial Cell-Derived Exosome-Mediated Delivery of Anti-miR-33a-5p to Cultured Macrophages. Curr. Issues Mol. Biol..

[52] Huang K., Pitman M., Oladosu O., Echesabal-Chen J., Vojtech L., Esobi I., Larsen J., Jo H., Stamatikos A. (2023). The Impact of MiR-33a-5p Inhibition in Pro-Inflammatory Endothelial Cells. Diseases.

[53] Peterson M.F., Otoc N., Sethi J.K., Gupta A., Antes T.J. (2015). Integrated systems for exosome investigation. Methods.

[54] Esobi I., Olanrewaju O., Echesabal-Chen J., Stamatikos A. (2022). Utilizing the LoxP-Stop-LoxP System to Control Transgenic ABC-Transporter Expression In Vitro. Biomolecules.

[55] Esobi I.C., Oladosu O., Echesabal-Chen J., Powell R.R., Bruce T., Stamatikos A. (2023). miR-33a Expression Attenuates ABCA1-Dependent Cholesterol Efflux and Promotes Macrophage-Like Cell Transdifferentiation in Cultured Vascular Smooth Muscle Cells. J. Lipids.

[56] Esobi I.C., Barksdale C., Heard-Tate C., Reigers Powell R., Bruce T.F., Stamatikos A. (2021). MOVAS Cells: A Versatile Cell Line for Studying Vascular Smooth Muscle Cell Cholesterol Metabolism. Lipids.

[57] Oladosu O., Esobi I.C., Powell R.R., Bruce T., Stamatikos A. (2023). Dissecting the Impact of Vascular Smooth Muscle Cell ABCA1 versus ABCG1 Expression on Cholesterol Efflux and Macrophage-like Cell Transdifferentiation: The Role of SR-BI. J. Cardiovasc. Dev. Dis..

[58] Fernando L., Echesabal-Chen J., Miller M., Powell R.R., Bruce T., Paul A., Poudyal N., Saliutama J., Parman K., Paul K.S. (2024). Cholesterol Efflux Decreases TLR4-Target Gene Expression in Cultured Macrophages Exposed to T. brucei Ghosts. Microorganisms.

[59] Schmittgen T.D., Livak K.J. (2008). Analyzing real-time PCR data by the comparative C(T) method. Nat. Protoc..

[60] Huang K., Garimella S., Clay-Gilmour A., Vojtech L., Armstrong B., Bessonny M., Stamatikos A. (2022). Comparison of Human Urinary Exosomes Isolated via Ultracentrifugation Alone versus Ultracentrifugation Followed by SEC Column-Purification. J. Pers. Med..

[61] Schneider C.A., Rasband W.S., Eliceiri K.W. (2012). NIH Image to ImageJ: 25 years of image analysis. Nat. Methods.

[62] White-Gilbertson S., Lu P., Esobi I., Echesabal-Chen J., Mulholland P.J., Gooz M., Ogretmen B., Stamatikos A., Voelkel-Johnson C. (2022). Polyploid giant cancer cells are dependent on cholesterol for progeny formation through amitotic division. Sci. Rep..

[63] Stamatikos A., Dronadula N., Ng P., Palmer D., Knight E., Wacker B.K., Tang C., Kim F., Dichek D.A. (2019). ABCA1 Overexpression in Endothelial Cells In Vitro Enhances ApoAI-Mediated Cholesterol Efflux and Decreases Inflammation. Hum. Gene Ther..

[64] Kong D.H., Kim Y.K., Kim M.R., Jang J.H., Lee S. (2018). Emerging Roles of Vascular Cell Adhesion Molecule-1 (VCAM-1) in Immunological Disorders and Cancer. Int. J. Mol. Sci..

[65] Singh V., Kaur R., Kumari P., Pasricha C., Singh R. (2023). ICAM-1 and VCAM-1: Gatekeepers in various inflammatory and cardiovascular disorders. Clin. Chim. Acta.

[66] Chong Z.X., Yeap S.K., Ho W.Y. (2021). Transfection types, methods and strategies: A technical review. PeerJ.

[67] Kalidasan V., Ng W.H., Ishola O.A., Ravichantar N., Tan J.J., Das K.T. (2021). A guide in lentiviral vector production for hard-to-transfect cells, using cardiac-derived c-kit expressing cells as a model system. Sci. Rep..

[68] Tietz S.M., Berghoff M. (2012). Gene silencing of MK2 in hard-to-transfect human U937 cells. J. Biomol. Tech..

[69] Rybakovsky E., Valenzano M.C., DiGuilio K.M., Buleza N.B., Moskalenko D.V., Harty R.N., Mullin J.M. (2019). Improving Transient Transfection Efficiency in a Differentiated, Polar Epithelial Cell Layer. J. Biomol. Tech..

[70] Dong S.X.M., Caballero R., Ali H., Roy D.L.F., Cassol E., Kumar A. (2020). Transfection of hard-to-transfect primary human macrophages with Bax siRNA to reverse Resveratrol-induced apoptosis. RNA Biol..

[71] Gonzalez-Romero E., Martinez-Valiente C., Garcia-Garcia G., Rosal-Vela A., Millan J.M., Sanz M.A., Sanz G., Liquori A., Cervera J.V., Vazquez-Manrique R.P. (2023). PCR-Based Strategy for Introducing CRISPR/Cas9 Machinery into Hematopoietic Cell Lines. Cancers.

[72] Berthier A., Staels B., Lefebvre P. (2021). An optimized protocol with a stepwise approach to identify specific nuclear receptor ligands from cultured mammalian cells. STAR Protoc..

[73] Churov A., Summerhill V., Grechko A., Orekhova V., Orekhov A. (2019). MicroRNAs as Potential Biomarkers in Atherosclerosis. Int. J. Mol. Sci..

[74] Feinberg M.W., Moore K.J. (2016). MicroRNA Regulation of Atherosclerosis. Circ. Res..

[75] Giral H., Kratzer A., Landmesser U. (2016). MicroRNAs in lipid metabolism and atherosclerosis. Best. Pract. Res. Clin. Endocrinol. Metab..

[76] Li Z., Zhao Y., Suguro S., Suguro R. (2023). MicroRNAs Regulate Function in Atherosclerosis and Clinical Implications. Oxid. Med. Cell Longev..

[77] Loyer X., Mallat Z., Boulanger C.M., Tedgui A. (2015). MicroRNAs as therapeutic targets in atherosclerosis. Expert. Opin. Ther. Targets.

[78] Lu Y., Thavarajah T., Gu W., Cai J., Xu Q. (2018). Impact of miRNA in Atherosclerosis. Arterioscler. Thromb. Vasc. Biol..

[79] Menghini R., Stohr R., Federici M. (2014). MicroRNAs in vascular aging and atherosclerosis. Ageing Res. Rev..

[80] Vartak T., Kumaresan S., Brennan E. (2022). Decoding microRNA drivers in atherosclerosis. Biosci. Rep..

[81] Price N.L., Goedeke L., Suarez Y., Fernandez-Hernando C. (2021). miR-33 in cardiometabolic diseases: Lessons learned from novel animal models and approaches. EMBO Mol. Med..

[82] Clerbaux L.A., Schultz H., Roman-Holba S., Ruan D.F., Yu R., Lamb A.M., Bommer G.T., Kennell J.A. (2021). The microRNA miR-33 is a pleiotropic regulator of metabolic and developmental processes in Drosophila melanogaster. Dev. Dyn..

[83] Price N.L., Fernandez-Tussy P., Varela L., Cardelo M.P., Shanabrough M., Aryal B., de Cabo R., Suarez Y., Horvath T.L., Fernandez-Hernando C. (2024). microRNA-33 controls hunger signaling in hypothalamic AgRP neurons. Nat. Commun..

[84] Rayner K.J., Sheedy F.J., Esau C.C., Hussain F.N., Temel R.E., Parathath S., van Gils J.M., Rayner A.J., Chang A.N., Suarez Y. (2011). Antagonism of miR-33 in mice promotes reverse cholesterol transport and regression of atherosclerosis. J. Clin. Investig..

[85] Marquart T.J., Wu J., Lusis A.J., Baldan A. (2013). Anti-miR-33 therapy does not alter the progression of atherosclerosis in low-density lipoprotein receptor-deficient mice. Arterioscler. Thromb. Vasc. Biol..

[86] Goedeke L., Salerno A., Ramirez C.M., Guo L., Allen R.M., Yin X., Langley S.R., Esau C., Wanschel A., Fisher E.A. (2014). Long-term therapeutic silencing of miR-33 increases circulating triglyceride levels and hepatic lipid accumulation in mice. EMBO Mol. Med..

[87] Price N.L., Zhang X., Fernandez-Tussy P., Singh A.K., Burnap S.A., Rotllan N., Goedeke L., Sun J., Canfran-Duque A., Aryal B. (2021). Loss of hepatic miR-33 improves metabolic homeostasis and liver function without altering body weight or atherosclerosis. Proc. Natl. Acad. Sci. USA.

[88] Karunakaran D., Thrush A.B., Nguyen M.A., Richards L., Geoffrion M., Singaravelu R., Ramphos E., Shangari P., Ouimet M., Pezacki J.P. (2015). Macrophage Mitochondrial Energy Status Regulates Cholesterol Efflux and Is Enhanced by Anti-miR33 in Atherosclerosis. Circ. Res..

[89] Ouimet M., Ediriweera H.N., Gundra U.M., Sheedy F.J., Ramkhelawon B., Hutchison S.B., Rinehold K., van Solingen C., Fullerton M.D., Cecchini K. (2015). MicroRNA-33-dependent regulation of macrophage metabolism directs immune cell polarization in atherosclerosis. J. Clin. Investig..

[90] Ouimet M., Ediriweera H., Afonso M.S., Ramkhelawon B., Singaravelu R., Liao X., Bandler R.C., Rahman K., Fisher E.A., Rayner K.J. (2017). microRNA-33 Regulates Macrophage Autophagy in Atherosclerosis. Arterioscler. Thromb. Vasc. Biol..

[91] Wu M.Y., Li C.J., Hou M.F., Chu P.Y. (2017). New Insights into the Role of Inflammation in the Pathogenesis of Atherosclerosis. Int. J. Mol. Sci..

[92] Baumer Y., McCurdy S., Weatherby T.M., Mehta N.N., Halbherr S., Halbherr P., Yamazaki N., Boisvert W.A. (2017). Hyperlipidemia-induced cholesterol crystal production by endothelial cells promotes atherogenesis. Nat. Commun..

[93] Cui X.B., Luan J.N., Dong K., Chen S., Wang Y., Watford W.T., Chen S.Y. (2018). RGC-32 (Response Gene to Complement 32) Deficiency Protects Endothelial Cells from Inflammation and Attenuates Atherosclerosis. Arterioscler. Thromb. Vasc. Biol..

[94] Liao J.K. (2013). Linking endothelial dysfunction with endothelial cell activation. J. Clin. Investig..

[95] Westerterp M., Tsuchiya K., Tattersall I.W., Fotakis P., Bochem A.E., Molusky M.M., Ntonga V., Abramowicz S., Parks J.S., Welch C.L. (2016). Deficiency of ATP-Binding Cassette Transporters A1 and G1 in Endothelial Cells Accelerates Atherosclerosis in Mice. Arterioscler. Thromb. Vasc. Biol..

[96] Vaisman B.L., Demosky S.J., Stonik J.A., Ghias M., Knapper C.L., Sampson M.L., Dai C., Levine S.J., Remaley A.T. (2012). Endothelial expression of human ABCA1 in mice increases plasma HDL cholesterol and reduces diet-induced atherosclerosis. J. Lipid Res..

[97] Kaur G., Dufour J.M. (2012). Cell lines: Valuable tools or useless artifacts. Spermatogenesis.

[98] Kheirolomoom A., Kim C.W., Seo J.W., Kumar S., Son D.J., Gagnon M.K., Ingham E.S., Ferrara K.W., Jo H. (2015). Multifunctional Nanoparticles Facilitate Molecular Targeting and miRNA Delivery to Inhibit Atherosclerosis in ApoE^-/-^ Mice. ACS Nano.

[99] Noveir S.D., Kerman B.E., Xian H., Meuret C., Smadi S., Martinez A.E., Johansson J., Zetterberg H., Parks B.A., Kuklenyik Z. (2022). Effect of the ABCA1 agonist CS-6253 on amyloid-beta and lipoprotein metabolism in cynomolgus monkeys. Alzheimers Res. Ther..

[100] Oram J.F., Lawn R.M., Garvin M.R., Wade D.P. (2000). ABCA1 is the cAMP-inducible apolipoprotein receptor that mediates cholesterol secretion from macrophages. J. Biol. Chem..

[101] Haidar B., Denis M., Krimbou L., Marcil M., Genest J. (2002). cAMP induces ABCA1 phosphorylation activity and promotes cholesterol efflux from fibroblasts. J. Lipid Res..

